# Supplementary material for: Metabolic control of enteroendocrine cell fate through a redox state sensor CtBP
Source: bioRxiv. 2025 Jul 2:2025.06.30.662346. Preprint. [Version 1] doi: 10.1101/2025.06.30.662346 (PMC12236740; doi:10.1101/2025.06.30.662346)
Supplement: Supplement 1 [file media-1.pdf]

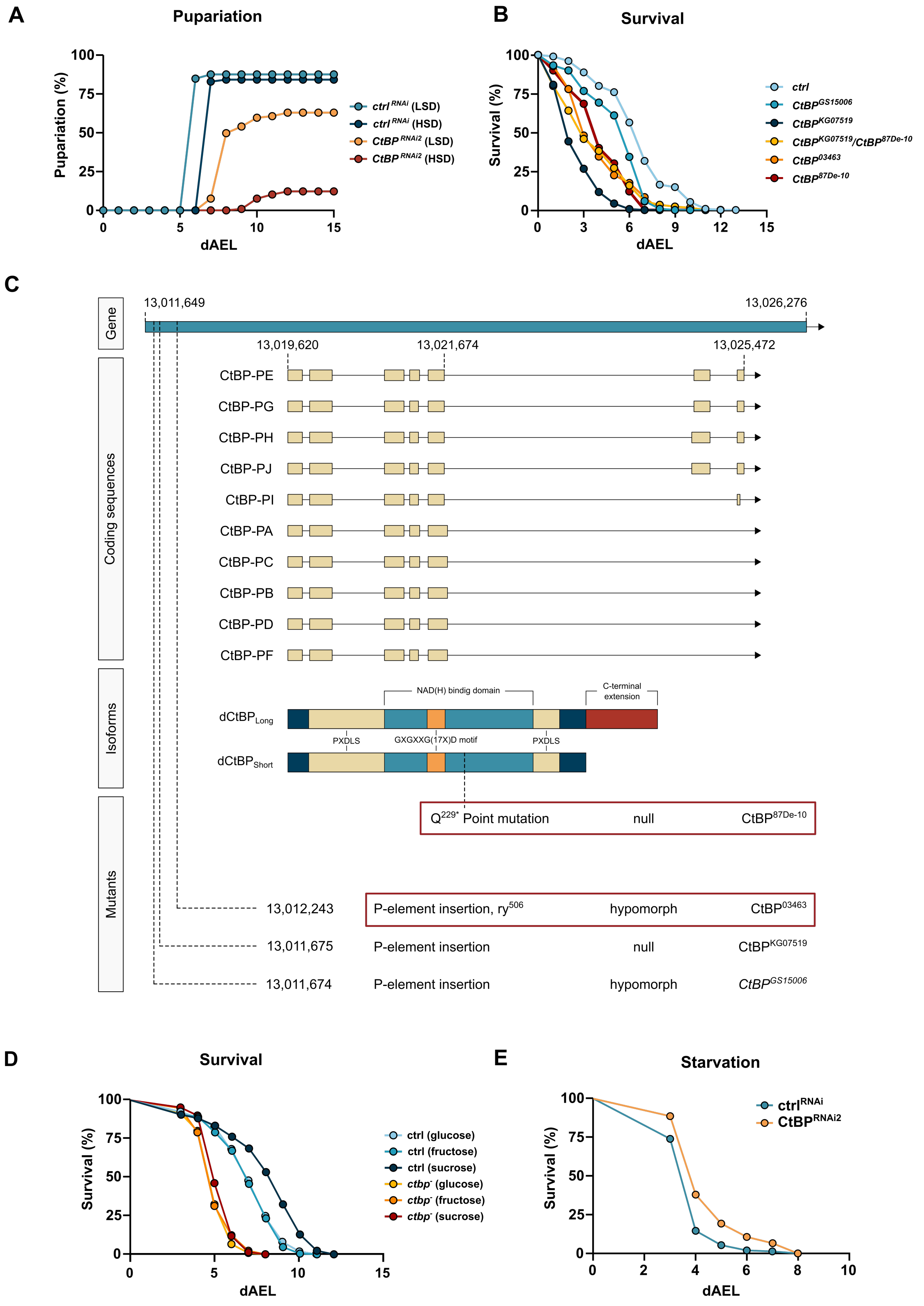

Figure S1. CtBP regulates *Drosophila* energy metabolism and is necessary for sugar tolerance.
(A) Whole-body knockdown of *CtBP* using an alternative RNAi line (*CtBP*^{RNAi2}) results in sugar intolerance. Pupariation rates for control and *CtBP*^{RNAi2} larvae on HSD were compared using the log-rank test ($\chi^2 = 71.9$, $P < 0.0001$). **(B)** Larvae carrying alternative *ctbp*$^{-}$ mutant alleles (different from those presented in Figure 1D) lived significantly shorter on 5% sucrose (adjusted for multiple comparisons log-rank test, $\chi^2 = 262.9$, $P < 0.0001$). **(C)** Genetic characteristics of *ctbp*$^{-}$ loss-of-function mutant alleles tested in this study. **(D)** *ctbp*$^{-}$ mutants display comparable sensitivity to 15% glucose and fructose (log-rank test, $\chi^2 = 0.67$, $P = 0.41$). Their survival on a 15% sucrose is modestly but significantly different compared to the monosaccharides (adjusted for multiple comparisons, log-rank test, $\chi^2 = 10.48$, $P = 0.0048$). Overall, *ctbp*$^{-}$ mutants exhibited significantly reduced survival compared to control animals (adjusted for multiple comparisons log-rank test, $\chi^2 = 584.3$, $P < 0.0001$) on a food with 15% dietary sugars. **(E)** Knockdown of *CtBP* using an alternative RNAi line (*CtBP*^{RNAi2}) increased starvation resistance of the larvae (log-rank test, $\chi^2 = 25.77$, $P < 0.0001$).

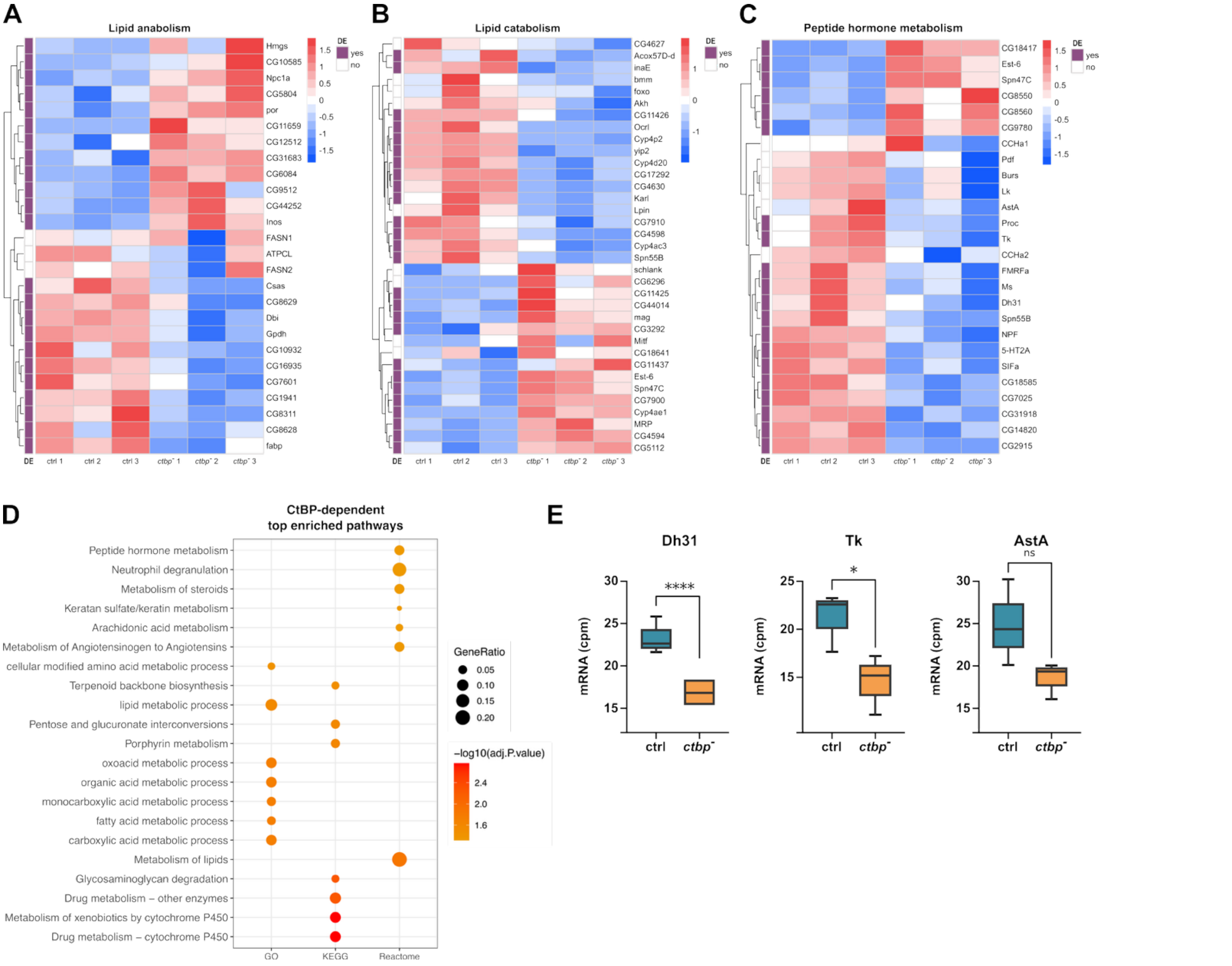

Figure S2. CtBP acts in enteroendocrine (EE) cells to control organismal metabolism.

ctbp-mutant larvae show dysregulation of genes involved in lipid anabolism **(A)** and catabolism **(B)** along with decreased expression and altered metabolism of peptide hormones **(C)**. The row-wise clustering was performed using Euclidean distance. Color key displays scaled log2 gene expression. (**D**) The top enriched pathways in *ctbp*-deficient larvae indicate a significant role for CtBP in peptide hormone and lipid metabolism. The color key represents the $\log_{10}$-adjusted P value, and circle size corresponds to the gene count in each pathway **(E)** Dh31, Tk, and AstA expression is impaired in *ctbp*-deficient larvae. *P = 0.0464;, **P = 0.011, ^{ns}P = 0.129. Adjusted P-values were calculated using Benjamini-Hochberg correction from differential expression analysis.

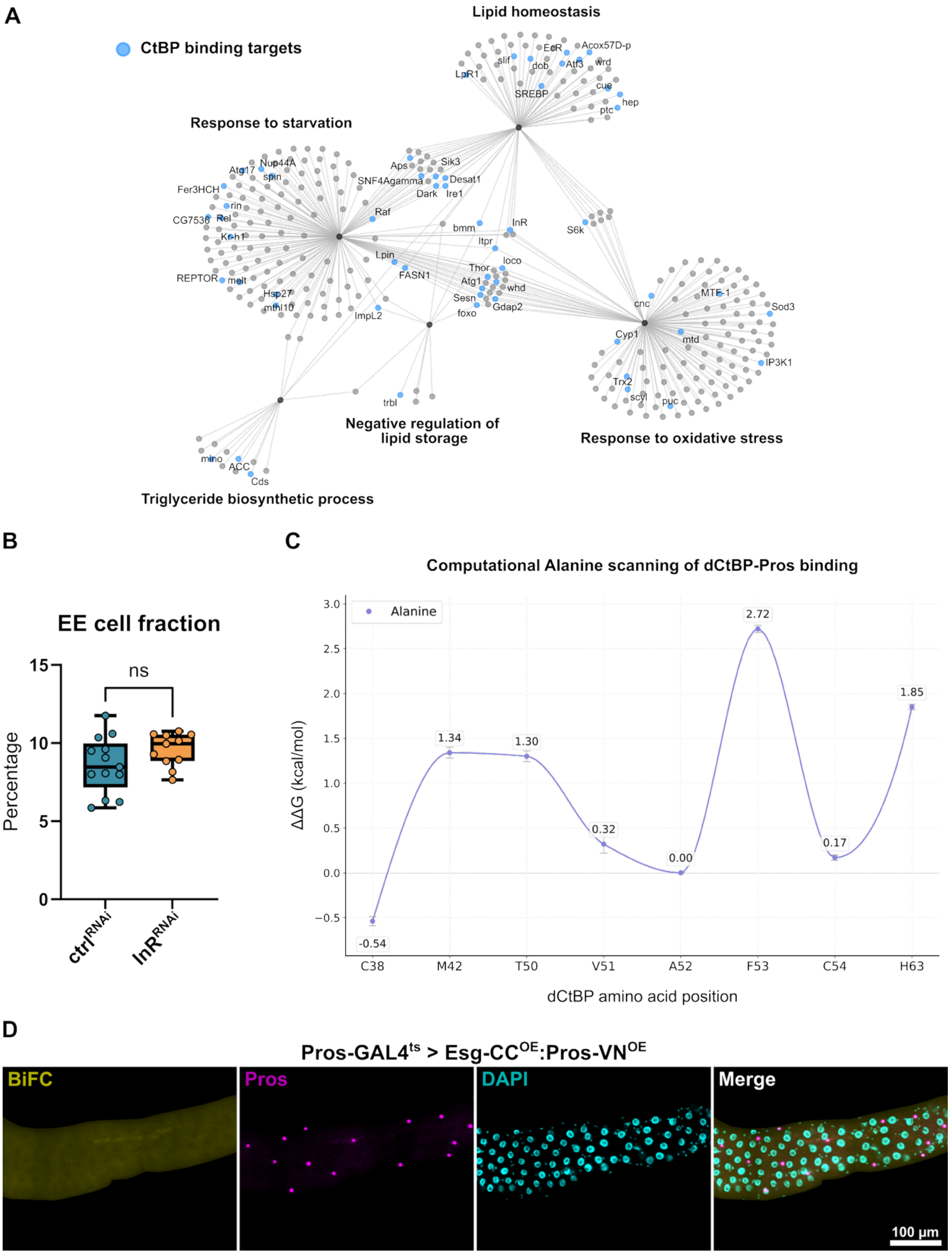

Figure S3. CtBP controls EE cell fate through Pros-dependent and independent pathways.
(A) CtBP genomic targets include regulators of lipid homeostasis and response to oxidative stress. CNET plot showing gene overlaps among selected pathways of CtBP targets identified by TaDa. Blue-colored nodes represent CtBP target genes within the pathway network. **(B)** Knockdown of *InR* in EE cells (Pros-GAL4>RNAi) does not affect the fraction of EE cells in larval intestine (unpaired t-test, ns indicates no significant difference in the studied parameter. **(C)** Computational scanning of dCtBP pocket residues for Pros binding reveals that the mutation of phenylalanine 53 to alanine (F53A) most significantly disrupts affinity with the Pros "ALSLV" motif ($\Delta\Delta G \approx 2.7$ kcal/mol). **(D)** Co-expression of Esg and CtBP BiFC fusion proteins in Prospero-driven EE cells does not produce fluorescence, serving as a negative control for the BiFC signal.
